# Pdcd4–Rictor Interaction Suppresses PFKFB3 to inhibit Tumorigenesis

**DOI:** 10.21203/rs.3.rs-9172690/v1

**Published:** 2026-04-22

**Authors:** Qing Wang, Yumeng Xin, Elham Zokaei, Ling Zeng, Dava Piecoro, Min Chen, Yanquan Zhang, Katie S. Yang, Chi Wang, Xiaoqi Liu, Hsin-Sheng Yang

**Affiliations:** 1 Department of Toxicology and Cancer Biology, College of Medicine, University of Kentucky, Lexington, Kentucky, USA; 2 Pathology and Laboratory Medicine, College of medicine, University of Kentucky, Lexington, Kentucky, USA; 3 Markey Cancer Center, College of Medicine, University of Kentucky, Lexington, Kentucky, USA

**Keywords:** Pdcd4, Rictor, mTORC2, PFKFB3

## Abstract

Programmed cell death 4 (Pdcd4) is a well-established tumor suppressor and inhibitor of protein translation. Although Pdcd4-mediated translational repression contributes to tumor suppression, emerging evidence suggests that Pdcd4 also exerts translation-independent functions. In this study, we found that Pdcd4 suppresses tumorigenesis through direct interaction with the rapamycin-insensitive companion of mTOR (Rictor), a core component of the mTORC2 complex. Using deletion mapping and site-directed mutagenesis, we defined the Rictor-binding domain of Pdcd4 and identified three critical residues, R105, K108, and R110, for this interaction. Co-immunoprecipitation and *in vitro* kinase assays demonstrated that Pdcd4 binding to Rictor disrupted mTORC2 complex assembly and inhibited its kinase activity. Reverse phase protein array analysis revealed that 6-phosphofructo-2-kinase/fructose-2,6-bisphosphatase 3 (PFKFB3), a key regulator of glycolysis, was markedly upregulated in Pdcd4-knockdown cells. Restoration of wild-type Pdcd4, but not a Rictor-binding–deficient mutant, reduced PFKFB3 protein abundance by promoting ubiquitin–proteasome–mediated degradation. Functionally, Pdcd4–Rictor interaction suppressed glycolytic activity and inhibited tumor cell proliferation in cultured cells and xenograft models. Consistent with these findings, non–small cell lung cancer (NSCLC) tissues exhibited significantly elevated protein levels of Rictor and PFKFB3 compared with adjacent normal tissues, with a positive correlation between their expression. Collectively, these results demonstrate that the translation-independent mechanism by which Pdcd4 disrupts mTORC2 signaling and downregulates PFKFB3 plays a critical role in suppressing NSCLC growth and glycolysis.

## INTRODUCTION

mTOR is a serine/threonine kinase that forms two functionally distinct complexes: mTORC1 and mTORC2. mTORC1, composed of mTOR, Raptor, mLST8, Deptor, and PRAS40, regulates cell growth primarily by controlling protein synthesis [1]. In contrast, mTORC2, consists of mTOR, Rictor, Sin1, mLST8, and Protor, phosphorylates Akt, serum glucocorticoid-regulated kinase 1 (SGK1), and protein kinase Cα (PKCα), thereby regulating cell proliferation, survival, apoptosis, and motility [2]. Rictor is a scaffolding protein, which is the key component in maintaining mTORC2 complex integrity and activity [3]. Genetic ablation of Rictor suppresses mTORC2 activity and subsequently attenuates cell proliferation, migration, and invasion [4, 5]. Conversely, Rictor up-regulation enhances mTORC2 activity, which in turn promotes cell proliferation and motility [6]. In mouse models, loss of Rictor-dependent mTORC2 activity decreases tumor growth and metastasis [7]. Importantly, Rictor amplification or Rictor overexpression is frequently observed across multiple cancer types, including lung cancer [8], and elevated Rictor expression is associated with poor prognosis or reduced survival rate [9]. In addition to binding with mTOR, Rictor may interact with other proteins to regulate tumorigenesis. For example, Rictor binding with integrin-linked kinase (ILK) mediates TGFβ-induced migration in breast cancer cells [10].

Several lines of evidence have shown that mTORC2 also plays an important role in regulating glycolysis by controlling the activation of key glycolytic enzymes [11]. For example, the mTORC2/Akt pathway activates hexokinase 2 (HK2) through phosphorylation at Thr473, enhancing the conversion of glucose to glucose-6-phosphate and thereby promoting glycolysis [12]. mTORC2 is also implicated in regulating the activity of 6-phosphofructo-2-kinase/fructose-2,6-bisphosphatase 3 (PFKFB3), another key enzyme in glycolysis [13]. PFKFB3 converts fructose-6-phosphate to fructose-2,6-bisphosphate to activate the rate-limiting enzyme, 6-phosphofructo-1-kinase (PFK-1) to boost glycolysis [14]. PFKFB3 expression has been reported to be up-regulated in lung cancer [15]. Inhibition or down-regulation of PFKFB3 in lung cancer cells induces cell apoptosis and inhibits tumor growth in nude mice [16]. Thus, PFKFB3 plays a critical role in both glycolysis and lung tumor growth.

Pdcd4 is a tumor suppressor whose expression is frequently downregulated in various cancers, including lung cancer [17]. Pdcd4 expression is primarily regulated through miR-21–mediated translation inhibition [18] and proteasome-mediated degradation [19]. Pdcd4 has been shown to inhibit tumor initiation and progression in various types of cancer cells [20]. Loss of Pdcd4 expression promotes cell proliferation and invasion [21, 22], whereas its overexpression suppresses these processes [23, 24]. Recent studies also suggest that Pdcd4 plays a critical role in enhancing chemosensitivity and overcoming drug resistance [25, 26]. Biochemically, Pdcd4 functions as a protein translation inhibitor. Up to date, several Pdcd4 translational targets have been identified, including stress-activated-protein kinase interacting protein 1 (Sin1) [21], p70S6K1 [27], Slug [28], XIAP [29], Bcl-XL [29], and c-Myb [30]. Suppression of the translation of these mRNAs by Pdcd4 occurs through either translation initiation factor 4A (eIF4A)-dependent mechanism, in which Pdcd4 inhibits the helicase activity of the eIF4A, or eIF4A-independent mechanism, whereby Pdcd4 directly binds to the mRNA to attenuate protein synthesis [26]. Despite its role in suppressing tumorigenesis by attenuating protein translation, Bera *et al.* reported that Pdcd4 interacts with Rictor to inhibit mTORC2 activity, thereby suppressing tumor cell invasion [31]. However, the mechanism by which Pdcd4 binds to Rictor and how this interaction influences mTORC2 activity and tumorigenesis remain unknown.

In this study, we identified the critical amino acids in Pdcd4 for binding with Rictor and demonstrated that Pdcd4-Rictor binding prevents the mTORC2 complex formation. We also provided evidence that Pdcd4-Rictor binding regulates glycolysis by affecting PFKFB3 protein abundance and NSCLC tumor growth. These findings reveal a new regulatory mechanism by which Pdcd4 inhibits NSCLC tumorigenesis through Rictor/mTORC2/PFKFB3 axis.

## MATERIALS AND METHODS

### Chemicals and reagents

Doxycycline hyclate (MedChem Express, HY-N0565B) was dissolved in sterile ddH_2_O, aliquoted, and stored at −80 °C. Cycloheximide (Sigma, 239763) was dissolved in DMSO, aliquoted, and stored at −20 °C. The wild-type, mutated, and methylated RBD peptides were synthesized by Shanghai Royobiotech Co. (China), purified to >95% by HPLC, and verified by mass spectrometry. The peptide was dissolved in water, aliquoted, and stored at −80 °C.

### Cell lines and culture conditions

All cell lines used in this study were purchased from the American Type Culture Collection. Lung cancer H1299 and H2172 cells were cultured in RPMI 1640 medium (HyClone, SH30027.01) supplemented with 10% FBS, 2 mM L-glutamine, and 100 U/mL penicillin-streptomycin; colorectal cancer HT-29 and HCT116 cells were cultured in McCoy’s medium (ThermoFisher Scientific, 16600082) with the same supplements; and HEK293 cells were cultured in high-glucose DMEM (HyClone, SH30022.01) supplemented with 10% FBS, 2 mM L-glutamine, and 100 U/mL penicillin-streptomycin. All cells were cultured at 37°C with 5% CO2 in a humidified incubator. All cell lines were routinely tested for mycoplasma contamination, and the cells passage number below 30 were used for this study.

### Plasmids and site-direct mutagenesis

The truncated Pdcd4 expressing plasmids were generated by PCR. The PCR products were subsequently cloned into EcoRI and NotI sites of the pCMV-HA-N vector (Takara Bio). For lentivirus Dox-inducible constructs, PCR products were cloned into MluI site of pCW57-RFP-P2A-MCS vector (Addgene, 78933) using In-Fusion^®^ Snap Assembly kit (Takara Bio). All constructs were sequenced to confirm in-frame fusion of pdcd4. The myc-Rictor expressing plasmid (11367) and PRMT5 expressing plasmid (HG11074-UT) was purchased from Addgene and Sino Biological, respectively.

### Immunofluorescence analysis of Pdcd4 and Rictor co-localization

The slides containing the normal human lung tissues were obtained from Biospecimen Procurement and Translational Pathology Shared Resource (BPTP SR) at the Markey Cancer Center, University of Kentucky. Tissue sections were de-waxed by heating at 60 °C, washed in xylene and re-hydrated through a graded series of ethanol and water. After antigen retrieval, tissues were blocked with normal goat serum. The tissues were then incubated with primary antibodies against Rictor (1:500 dilution, Millipore Sigma, PLA0309) and Pdcd4 (1:1500 dilution) followed by anti-mouse secondary antibody conjugated with Alexa Fluor^™^ 488 (ThermoFisher, A-11001) and anti-rabbit secondary antibody conjugated with Alexa Fluor^™^ 568 (ThermoFisher, A-11011), respectively. The Pdcd4 antibody was generated in-house as described previously [32].

### Immunoprecipitation analysis

Cells (HEK293, HCT116, or H1299) were transfected with Pdcd4 or Rictor expressing plasmid using PolyJet transfection reagent (SignaGen Laboratories, SL100688). After 48 h, cells were harvested and lysed in immunoprecipitation (IP) lysis buffer containing 40 mM HEPES, pH 7.4, 120 mM NaCl, 2 mM EDTA, 0.3% CHAPS, 10 mM pyrophosphate, 10 mM glycerophosphate, and 50 mM NaF, supplemented with 1 protease inhibitor tablet per 10 ml of buffer (ThermoFisher Scientific, PIA32953). Six hundred to eight hundred micrograms of cell lysates were incubated for 1 h at room temperature under the following conditions: with anti-HA magnetic beads (ThermoFisher Scientific, PI88837) for Pdcd4 immunoprecipitation of Rictor; with anti-Myc antibody (Santa Cruz, sc-40) and Protein G magnetic beads (ThermoFisher Scientific, PI88847) for Rictor immunoprecipitation of Pdcd4; with anti-mTOR antibody (Cell Signaling, 2972) and Protein G magnetic beads for mTOR complex immunoprecipitation; or with anti-His antibody (Cell Signaling, 12698) for ubiquitinated PFKFB3 immunoprecipitation. For endogenous immunoprecipitation, 30 μl of Rictor conjugated Sepharose^®^ Beads (Cell signaling, 5379) was incubated with 600 μg of H1299 cell lysates for 1 h at room temperature. After washing with lysis buffer, the precipitated complexes were eluted with 30 μl of SDS sample buffer.

### Transient transfection

Cells (2.5 ×10^5^) were seeded on a 60 mm plate and transfected with 2.5 μg of plasmid expressing Pdcd4 (full-length, truncated, or mutated), Rictor, or PFKFB3 using PolyJet transfection reagent. Cells were subsequently used for Western blot or proliferation assays.

### Western blot analysis

Cells were lysed in lysis buffer containing 50 mM Tris-HCl (pH 7.5), 150 mM NaCl, 1% Nonidet P40, 0.5% sodium deoxycholate, supplemented with 1X Halt^™^ Protease and Phosphatase Inhibitor Cocktail (ThermoFisher Scientific, 78442). The protein concentration of cell lysates was determined with BCA Protein Assay Kit (ThermoFisher Scientific, 23227).

The immuno-precipitated complexes or 10-30 μg of cell lysate were loaded onto 4-20% SurePAGE (GenScript, M00656). Immunoblot analysis and quantification of band intensity were performed as described previously [32]. The following primary antibodies were used for Western blot: anti-Actin (1:4000 dilution, Santa Cruz, sc-47778), anti-pan Akt (1:2000 dilution, Cell Signaling, 4691), anti-phospho-Akt(Ser473) (1:2000 dilution, Cell Signaling, 4060), anti-GAPDH (1:4000 dilution, Santa Cruz, sc-47724), anti-HA tag (1:2000 dilution, Cell signaling, 3724), anti-His (1:1000 dilution, Cell Signaling, 12698), anti-mTOR (1:1000 dilution, Cell signaling, 2972), anti-PFKFB3 (1:10000 dilution, Bethyl, A304-249A), anti-Pdcd4 (1:4000 dilution), anti-Raptor (1:1000 dilution, Cell signaling, 2280), anti-Rictor (1:1000 dilution, Cell signaling, 2114), anti-phospho-S6K(Thr389) (1:1000 dilution, Cell signaling, 9234), anti-S6K (1:1000 dilution, Cell Signaling, 2708), and anti-Sin1 (1:5000 dilution, Novus Biologicals, NB110-40424).

### Reverse phase protein array (RPPA)

RPPA was performed by the Center for Environmental and Systems Biochemistry (Redox Metabolism Shared Resource Facility, University of Kentucky) as previously described [33]. Briefly, cell lysates (0.5 mg/ml) of HCT116 cells expressing shLacZ (control) or shPdcd4 (Pdcd4 knockdown) were printed as spots onto nitrocellulose membrane pads using microarray printer (ArrayJet). After quantification of total protein per spot using an Azure RPPA staining kit (VWR, AC2233), slides were scanned at 700 nm emission wavelength using an InnoScan 710 AL Microarray Scanner (Innopsys). Slides were then immunoblotted with ELISA grade primary antibodies overnight at 4 °C, followed by incubation with AzureSpectra 800 fluorescent secondary antibodies for 1 h at room temperature, and subsequently scanned at 800 nm emission wavelength using the InnoScan 710 AL.

### Tissue microarray and immunohistochemical staining

The tissue microarray (TMA) was generated by the BPTP SR at the Markey Cancer Center, University of Kentucky. Eighty-seven formalin-fixed, paraffin-embedded lung adenocarcinoma tumors and paired adjacent normal specimens were reviewed by a pathologist, and their pathological characteristics are listed in Supplementary Table I. Tumor and normal tissue cores (2 mm in diameter) were incorporated into the array, and 5-μm-thick sections were subsequently cut and mounted onto glass slides for immunohistochemical analysis.

The immunohistochemical staining for TMA and tumor slides from H1299 xenograft was performed using ImmPRESS^®^ Excel Amplified Polymer Kit (Vector laboratories, MP-7601). The tissue sections were de-waxed by heating at 60°C, washed in xylene and re-hydrated through a graded series of ethanol and water. The antigen retrieval and targeted proteins were visualized following the manufacturer’s protocol. The following antibodies were used for immunohistochemical staining in this study: anti-Rictor (1:1000 dilution, Bethyl, A304-329A), anti-PFKFB3 (1:500 dilution, ThermoFisher, PA5-21931), anti-Ki67 (1:1500 dilution, cell Signaling, 12202), and cleaved caspase 3 (1:500 dilution, Cell Signaling, 9664).

### mTORC2 kinase assay

The mTORC2 kinase assay was performed as described previously [21].

### Knockdown of Rictor or Pdcd4

H1299 cells (2 × 10^5^) were transfected with 110 pmol of *rictor* #1 siRNA (ThermoFisher, AM16708) and *rictor* #2 siRNA (Santa Cruz, sc-61479) using INTERFERin *in vitro* siRNA transfection reagent (Polyplus, 101000028) as instructed by the manufacturer’s protocol. After 48 h, cells were harvested for immunoblot analyses. For Pdcd4 knockdown cells, HCT116 cells were transduced with lentivirus containing the *lacZ* shRNA (control) or *pdcd4* shRNA as previously described [28].

### Cell proliferation and clonogenic assays

The XTT Cell Viability Assay Kit (Biotium, 30007) was used for cell proliferation assays as described previously [34]. Twenty-four hours post-transfection of Pdcd4 and/or PFKFB3 expressing plasmids, 1000 cells per well were seeded onto a 96-well plate. At the time points indicated in figures, 25 μl of activated XTT was added to each well and incubated at 37°C for 4 h. Absorbance at 470 nm was then measured using the BioTek Cytation 5 Cell Imaging Multimode Reader (Agilent). For clonogenic assays, 500 cells were seeded onto a six-well plate and subsequently cultured for 7 days. The colonies were visualized by staining with 1% (w/v) crystal violet.

### *In vivo* xenograft study

Athymic nude mice (Crl:NU(NCr)-Foxn1^nu^, strain code:490) were purchased from Charles River Laboratories at 4-5 weeks of age. The mice were housed under pathogen-free conditions with commercial diet, water ad libitum, and 12 h light/12 h dark cycle. The experimental protocol was approved by IACUC of the University of Kentucky based on the NIH guidelines. Six mice (3 males and 3 females) were subcutaneously injected with 1X10^6^ H1299 cells expressing either the empty Dox-inducible vector (control), Dox-inducible Pdcd4(1–156) construct, or Dox-inducible Pdcd4(1–156)(3A) construct. One day after tumor cells injection, the mice were orally administered doxycycline hyclate (5mg/kg) daily until the end of the experiment. Tumor size was evaluated 10 days after injection using the following formula: volume = (length × width^2^)/0.52. At the end of experiment, the mice were euthanized, and the tumor weight was measured.

### Correlation analysis of *rictor* and *pfkfb3* mRNA levels in TCGA PanCancer Data

For correlation analysis of *rictor* and *pfkfb3* mRNA expression, log-transformed mRNA expression z-scores from lung adenocarcinoma patients in The Cancer Genome Atlas (TCGA) PanCancer study (n= 510) [35] and OncoSG study (n=169) [36] were downloaded from cBioPortal (www.cbioportal.org). The Spearman’s correlation coefficient was used to quantify the associations between Rictor and PFKFB3 expression levels.

### Statistical analysis

The levels of Rictor and PFKFB3 protein in malignant and adjacent normal tissues were analyzed by Wilcoxon matched-pairs signed rank test. Spearman’s correlation coefficient was used to quantify the correlation between Rictor and PFKFB3 expressions. Kaplan–Meier curve and log-rank test were used to compare overall survival between groups. Band intensities from immunoblots were analyzed using a one-sample *t*-test, comparing control and treated groups by testing whether the mean relative fold change was equal to one. For other experiments, the two-sample *t*-test assuming equal variances was used to compare experimental groups. Data are expressed as mean ± s.d., and *P* < 0.05 was considered statistically significant. The number of replicates is indicated in the figure legends.

## RESULTS

### Pdcd4 binds with Rictor

**Fig. 1:**
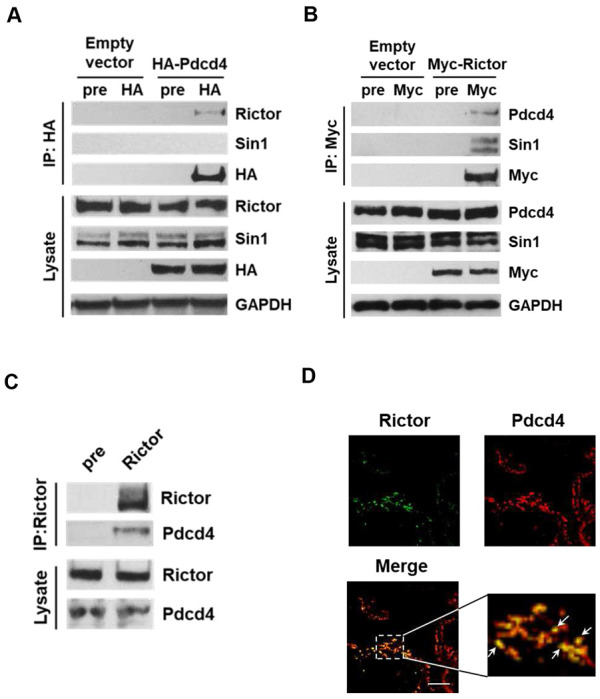
Pdcd4 binds with Rictor. **A,** Immunoprecipitation of endogenous Rictor using HA-tagged Pdcd4. The HA-tagged Pdcd4 plasmid was transiently transfected into HEK293 cells and immunoprecipitated with anti-HA antibody. Pre, rabbit preimmune serum. **B,** Reciprocal immunoprecipitation of endogenous Pdcd4 by myc-tagged Rictor. The myc-tagged Rictor plasmid was transiently transfected into HEK293 cells and immunoprecipitated with an anti-myc antibody. Pre, rabbit preimmune serum. **C,** Immunoprecipitation of the endogenous Pdcd4-Rictor complex from H1299 cells using an anti-Rictor antibody. **D,** Co-localization of Pdcd4 and Rictor in normal lung tissue. Rictor (green) was detected using anti-Rictor antibody followed by anti-mouse secondary antibody conjugated with Alexa Fluor^™^ 488; Pdcd4 (red) was detected using anti-Pdcd4 antibody followed by anti-rabbit secondary antibody conjugated with Alexa Fluor^™^ 568.

A previous study suggested that Pdcd4 may interact with Rictor and inhibit mTORC2 activity [31]. To confirm whether Rictor serves as a binding partner for Pdcd4, HA-tagged Pdcd4 (HA-Pdcd4) was over-expressed in HEK293 cells. Immunoprecipitation with an anti-HA antibody revealed that endogenous Rictor co-precipitated with HA-Pdcd4 (Fig. 1A). In parallel, over-expression of myc-tagged Rictor (myc-Rictor) led to co-immunoprecipitation with endogenous Pdcd4 (Fig. 1B). Notably, Pdcd4 did not co-immunoprecipitate with Sin1, a distinct component of the mTORC2 complex (Fig. 1A), indicating that Pdcd4 specifically binds to Rictor rather than interacting with the entire mTORC2 complex. Moreover, immunoprecipitation of endogenous Rictor with an anti-Rictor antibody also pulled down endogenous Pdcd4 (Fig. 1C), reinforcing the existence of a specific interaction between Pdcd4 and Rictor. To further assess the physiological relevance of Pdcd4-Rictor binding, normal lung tissues were incubated with anti-Rictor and anti-Pdcd4 antibodies, followed by anti-mouse secondary antibody conjugated with Alexa Fluor^™^ 488 (green) and anti-rabbit secondary antibody conjugated with Alexa Fluor^™^ 568 (red), respectively. The observed co-localization of Pdcd4 and Rictor in normal lung tissues (Fig. 1D) demonstrates that their interaction occurs *in vivo* under physiological conditions. Collectively, these results establish Rictor as a bona fide binding partner of Pdcd4.

### Identification of Rictor binding domain on Pdcd4

**Fig. 2:**
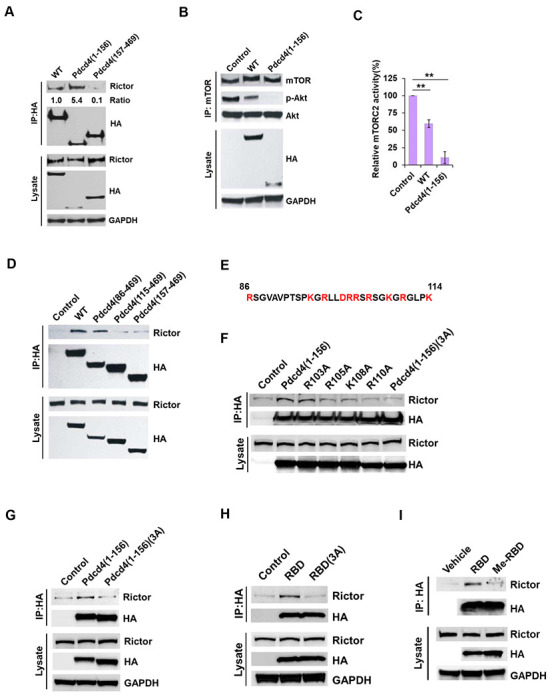
Characterization of the Rictor binding domain in Pdcd4. **A,** Pdcd4(1-156) binds with Rictor. HEK293 cells were transfected with HA-tagged full-length (WT), Pdcd4(1-156) and Pdcd4(157-469) plasmids and immunoprecipitated with an anti-HA antibody. **B and C,** Pdcd4(1-156) inhibits mTORC2 kinase activity. B, representative images of mTORC2 kinase assay; C, quantification of mTORC2 kinase activity. The ratio of p-Akt/precipitated mTOR in control cells was set to 100%. Data were analyzed by a one-sample *t*-test (n=3; mean±s.d.; **p < 0.01). **D,** Deletion analyses to map the Rictor binding domain. HA-tagged full-length (WT) and various Pdcd4 deletion mutant plasmids were transfected into HEK293 cells and immunoprecipitated with an anti-HA antibody. **E,** Amino acid sequence of RBD. The charged amino acid residues are in red. **F,** Site-specific mutagenesis analysis to identify critical residues for Rictor binding. Various HA-tagged Pdcd4 mutant plasmids were transfected into HEK293 cells and immunoprecipitated with an anti-HA antibody. **G,** Pdcd4(1-156)(3A) does not bind with Rictor. HA-tagged Pdcd4(1-156) and Pdcd4(1-156)(3A) plasmids were transfected into H1299 cells and immunoprecipitated with anti-HA antibody. **H,** RBD but not RBD(3A) peptide binds with Rictor. HA-tagged RBD and RBD(3A) peptides were incubated with H1299 cell lysate and immunoprecipitated with an anti-HA antibody. **I,** Methylated RBD peptide does not bind with Rictor. HA-tagged RBD and methylated RBD (Me-RBD) peptides were incubated with H1299 cell lysate and immunoprecipitated using an anti-HA antibody. All immunoprecipitation assays were followed by Western blot analysis with the indicated antibody.

To identify the specific region of Pdcd4 responsible for binding to Rictor, the Pdcd4 was divided into two fragments: amino acids 1–156 [Pdcd4(1–156)] and amino acids 157–469 [Pdcd4(157–469)]. The Pdcd4(157-469) fragment is known to inhibit protein translation [37], while the function of the Pdcd4(1-156) has not been well characterized. HA-tagged full-length (WT), Pdcd4(1-156), and Pdcd4(157-469) plasmids were transfected into HEK239 cells and subjected to immunoprecipitation using an anti-HA antibody. As shown in Fig. 2A, Pdcd4(1-156), but not Pdcd4(157-469) co-precipitated with endogenous Rictor. In addition, the Pdcd4(1-156) fragment was sufficient to inhibit mTORC2 kinase activity (Figs. 2B and C). To pinpoint the exact Rictor-binding site on Pdcd4, a series of HA-tagged Pdcd4 deletion mutants were generated and transfected into HEK293 cells. Both the full-length Pdcd4 (WT) and the Pdcd4(86–469) fragment were able to immunoprecipitate with Rictor (Fig. 2D). Since Pdcd4(1-156) and Pdcd4(86-469), but not Pdcd4(115-469) (Figs. 2A and D) bound to Rictor, the Rictor binding domain (RBD) was localized to amino acid 86-114. Sequence analyses of this region revealed 10 charged residues, including 6 Arginine (R), 3 Lysine (K), and 1 Aspartic acid (D) (Fig. 2E). As charged residue can form an ionic interaction with other proteins, we replaced positively the charged amino acid with alanine. As shown in Fig. 2F, mutations at positions 105, 108, and 110 markedly reduced Rictor binding to background levels, similar to empty vector-transfected controls, indicating that these three residues are crucial for Rictor binding. The triple mutant [Pdcd4(1-156)(3A)] also reduced Rictor binding to the background-level (Fig. 2F). Consistent results were observed in lung cancer H1299 cells expressing empty vector, Pdcd4(1–156), and Pdcd4(1–156)(3A) (Fig. 2G).

To further examine this interaction, we synthesized an HA-tagged RBD peptide and an RBD peptide with alanine substitution at positions 105, 108, and 110 [RBD(3A)]. As shown in Fig. 2H, the RBD peptide, but not RBD(3A), was able to bind Rictor. Additionally, previous studies by Power *et al.* showed that Pdcd4 can be methylated at R110 by protein arginine methyltransferase 5 (PRMT5) [38]. To test whether methylation of Pdcd4 at R110 interferes with Rictor binding, an HA-tagged RBD peptide with a di-methylated arginine at position 110 (Me-RBD) was synthesized and immunoprecipitated with Rictor from H1299 cell lysates. As shown in Fig. 2I, Me-RBD displayed markedly reduced binding to Rictor compared to the unmodified RBD peptide. Similar findings were observed when PRMT5 cDNA was co-transfected with the HA-tagged Pdcd4(1–156) and the complexes were immunoprecipitated with an anti-HA antibody. (Supplementary Fig. 1). These results collectively demonstrate the critical role of residues R105, K108, and R110 in Pdcd4’s binding to Rictor.

### Pdcd4-Rictor binding prevents mTORC2 complex formation

**Fig. 3:**
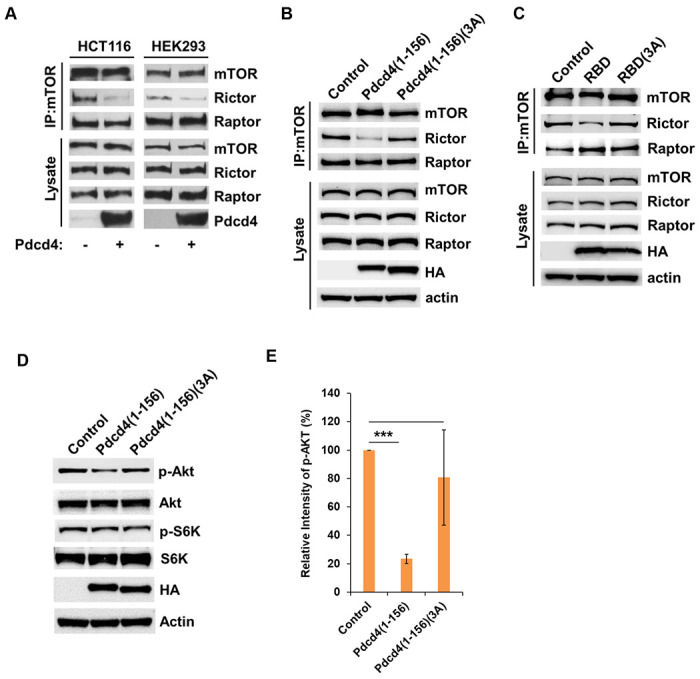
Pdcd4-Rictor binding prevents mTORC2 complex formation. **A,** Pdcd4 blocks Rictor binding to mTOR. mTORC1 and mTORC2 complexes were precipitated with anti-mTOR antibody from HCT116 cells and HEK293 cells overexpressing Pdcd4, followed by Western blot analysis with the indicated antibody. **B,** Pdcd4(1-156) prevents Rictor from binding to mTOR. Pdcd4(1-156) and Pdcd4(1-156)(3A) were over-expressed in H1299 cells, followed by immunoprecipitation using an anti-mTOR antibody and Western blot analysis with the indicated antibodies. **C,** RBD peptide blocks Rictor binding to mTOR. RBD and RBD(3A) peptide were incubated with H1299 cell lysates, followed by immunoprecipitation using an anti-mTOR antibody and Western blot analysis with the indicated antibodies. **D and E,** Pdcd4(1-156) but not Pdcd4(1-156)(3A) inhibits Akt phosphorylation at Ser473. Pdcd4(1-156) and Pdcd4(1-156)(3A) were overexpressed in H1299 cells followed by Western blot analysis with the indicated antibodies. D, representative immunoblots images; E, quantification of phospho-Akt(Ser473) band intensities. The ratio of p-Akt/Actin in control cells was set to 100%. One sample *t*-test was used for comparing the control and Pdcd4(1-156) groups (n=3; mean±s.d.; ###*P* < 0.001). Two sample *t*-test was used for comparing the Pdcd4(1-156) and Pdcd4(1-156)(3A) groups (n=3; mean±s.d.; *P < 0.05).

Our data demonstrated that full-length Pdcd4 and Pdcd4(1-156) fragment inhibited mTORC2 kinase activity (Fig. 2B and C), suggesting that Pdcd4-Rictor binding may affect mTORC2 complex formation. To address this, we performed immunoprecipitation using an mTOR antibody to capture mTORC1 and mTORC2 complexes from cell lysates. In both HCT116 and HEK293 cells, expression of full-length Pdcd4 led to decreased association of Rictor with mTOR compared to cells transfected with an empty vector (Fig. 3A). Moreover, the expression of Pdcd4(1-156) or addition of RBD peptide to cell lysates also reduced the amount of Rictor associated with mTOR. In contrast, the mutant forms Pdcd4(1-156)(3A) and RBD(3A) did not affect this association (Fig. 3B and C). These findings indicate that Pdcd4 binding to Rictor disrupts the formation of the mTORC2 complex. Notably, neither full-length Pdcd4, Pdcd4(1–156), nor RBD interfered the Raptor-mTOR interaction, suggesting that Pdcd4 does not inhibit mTORC1 activity (Fig. 3B and C). Consistent with this concept, H1299 cells expressing Pdcd4(1–156) showed reduced Akt phosphorylation at Ser473 (a substrate of mTORC2), while the phosphorylation of S6K at Thr389 (a substrate of mTORC1) remained unaffected (Fig. 3D and E).

### Pdcd4-Rictor interaction inhibits glycolysis via promoting PFKFB3 degradation

**Fig. 4:**
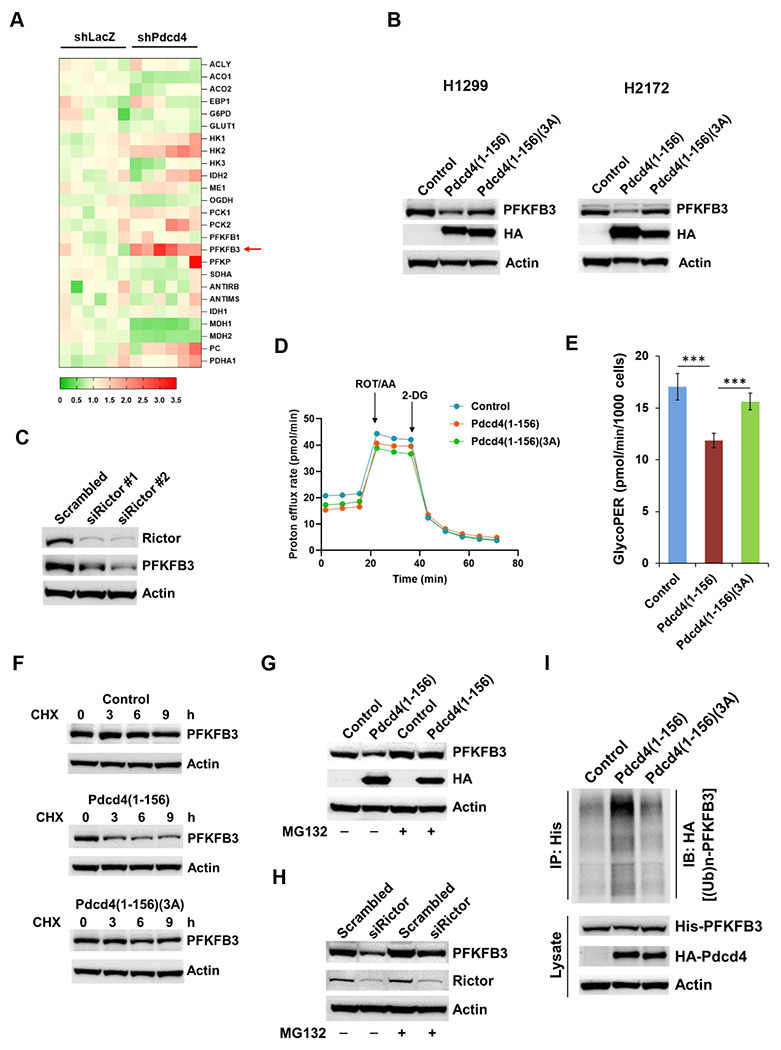
Pdcd4-Rictor interaction reduces PFKFB3 protein abundance. **A,** RPPA assay. The heat map shows alterations in proteins associated with glycolysis and the TCA cycle in both control and Pdcd4-knockdown cells. The red arrow indicates PFKFB3. **B,** Pdcd4(1-156) but not Pdcd4(1-156)(3A) reduces the PFKFB3 protein level in NSCLC cells. HA-tagged Pdcd4(1-156) and Pdcd4(1-156)(3A) plasmids were overexpressed in H1299 and H2172 cells and analyzed by Western blot using the indicated antibodies. **C,** Knockdown of Rictor reduces PFKFB3 protein level. The *rictor* siRNAs were transfected into H1299 cells and analyzed by Western blot with the indicated antibodies. **D,** Seahorse glycolytic rate assay. H1299 cells were transfected with either an empty vector (control), Pdcd4(1-156), or Pdcd4(1-156)(3A) plasmid, and then subjected to Seahorse glycolytic rate assay. The extracellular acidification rate (ECRA) and mitochondrial oxygen consumption rate (OCR) was measured following sequential injection of rotenone/antimycin A (Rot/AA) and 2-deoxy-D-glucose (2-DG) and then converted to proton efflux rate. **E,** Overexpression of Pdcd4(1-156) lowers glycolysis. Quantification of glycolysis rate from Seahorse glucose glycolytic rate in panel D. **F,** Pdcd4(1-156) but not Pdcd4(1-156)(3A) decreases PFKFB3 protein stability. H1299 cells were transfected with the empty vector (control), Pdcd4(1-156), and Pdcd4(1-156)(3A) plasmids, followed by treatment of cycloheximide (10 μg/ml). The cells were harvested at various time points and analyzed by immunoblotting with the indicated antibodies. **G and H,** Pdcd4(1-156) expression or Rictor knockdown enhances PFKFB3 degradation. H1299 cells with Pdcd4(1-156) overexpression or Rictor knockdown were treated with or without the proteasome inhibitor, MG132 (10 μM) for 4 h, then analyzed by immunoblotting with the indicated antibodies. **I,** Pdcd4(1-156) promotes PFKFB3 ubiquitination. HA-tagged Pdcd4(1-156) or Pdcd4(1-156)(3A) was co-expressed with His-tagged PFKFB3 and HA-tagged ubiquitin expressing plasmids, immunoprecipitated with anti-His antibody, and analyzed by Western blotting with the indicated antibodies.

Previous studies have suggested that mTORC2 may regulate glycolysis [39], and since our data indicate that Pdcd4 inhibits mTORC2 activity (Fig. 2A–C), we sought to determine whether Pdcd4 affects glycolysis. We first examined whether Pdcd4 alters the protein level of key enzymes involved in the glycolysis pathway. Cell lysates from Pdcd4 knockdown (shPdcd4) and control (shLacZ) cells were analyzed using RPPA to profile the abundance of proteins associated with glycolysis and the TCA cycle. We found that the protein levels of HK2 and PFKFB3 were significantly up-regulated in Pdcd4 knockdown cells, whereas aconitase 1 (ACO1), malate dehydrogenase 1 (MDH1), MDH2, and oxoglutarate dehydrogenase (OGDH) were downregulated (Fig. 4A and supplementary Fig. 2). Because HK2 and PFKFB3 function in glycolysis, and ACO1, MDH1, MDH2, and OGDH are components of the TCA cycle, these findings indicate that Pdcd4 knockdown enhances glycolysis while suppressing TCA cycle activity. This suggests that loss of Pdcd4 contributes to the Warburg effect, a metabolic shift commonly observed in cancer cells. As PFKFB3 exhibited the greatest increase in protein levels in the RPPA analysis, we therefore focused on how Pdcd4 influences PFKFB3 expression. The results showed that Pdcd4(1-156), but not the Rictor-binding-deficient mutant Pdcd4(1-156)(3A), reduced PFKFB3 protein abundance in lung cancer H1299 and H2172 cells (Fig. 4B). Additionally, knockdown of Rictor was performed to assess whether mTORC2 regulates PFKFB3 protein levels. As shown in Fig. 4C, Rictor knockdown also led to a reduction in PFKFB3 protein, indicating that Pdcd4/mTORC2 axis plays a role in regulating PFKFB3 protein abundance. In agreement with the reduced PFKFB3 level, the Seahorse glycolysis analysis showed that Pdcd4(1-156), but not Pdcd4(1-156)(3A), significantly suppressed the glycolysis in H1299 cells (Figs. 4D and E). These findings highlight the importance of Pdcd4-Rictor interaction in suppressing glycolysis in lung cancer cells.

To investigate the mechanism of how Pdcd4 down-regulates PFKFB3 protein abundance, cycloheximide chase experiments were conducted to assess protein stability of PFKFB3 protein in cells expressing Pdcd4(1-156) compared to those expressing Pdcd4(1-156)(3A) or empty vector. The results showed that Pdcd4(1-156) expression shortened the half-life of PFKFB3, indicating that the Pdcd4-Rictor interaction decreases PFKFB3 protein stability (Fig. 4F). Previous studies have reported that PFKFB3 can be degraded through ubiquitination-mediated pathways [40]. To determine whether the reduction of PFKFB3 by Pdcd4(1-156) involves the ubiquitin-proteasome system, H1299 cells expressing Pdcd4(1-156) or empty vector were treated with the proteasome inhibitor MG132. As shown in Fig. 4G, MG132 treatment resulted in an increased PFKFB3 protein level in Pdcd4(1-156) expressing cells. Similarly, Rictor knockdown cells exhibited elevated PFKFB3 levels following MG132 treatment (Fig. 4H). Furthermore, cells expressing Pdcd4(1-156) showed increased PFKFB3 ubiquitination compared to control cells or those expressing Pdcd4(1-156)(3A) (Fig. 4I). These findings collectively indicate that Pdcd4-Rictor binding promotes proteasome-mediated degradation of PFKFB3, thereby decreasing its protein abundance.

### Pdcd4-Rictor interaction suppresses NSCLC cell proliferation and tumorigenesis

**Fig. 5:**
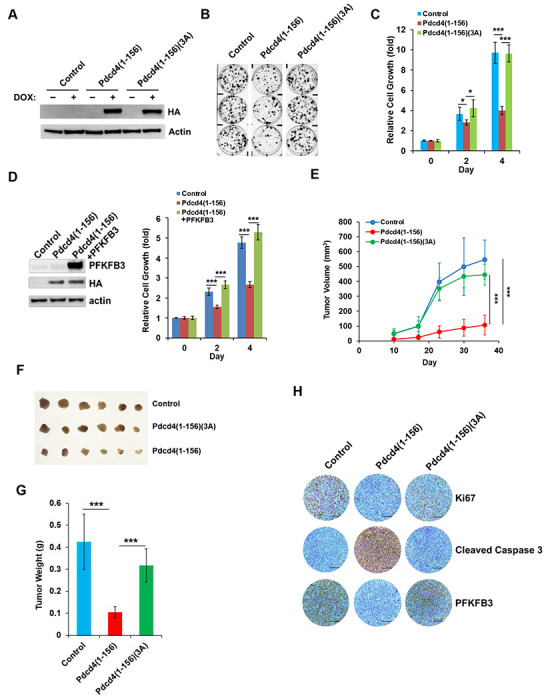
Pdcd4(1-156) but not Pdcd4(1-156)(3A) attenuates cell proliferation and tumor growth. **A,** H1299 cells expressing Dox-inducible Pdcd4(1-156) or Pdcd4(1-156)(3A) were treated with doxycycline (0.5 μg/ml) for 2 days and analyzed by Western blot using the indicated antibodies. **B,** Overexpression of Pdcd4(1-156) inhibits colony formation. H1299 cells expressing Dox-inducible Pdcd4(1-156) or Pdcd4(1-156)(3A) were seeded onto a 6-well plate and treated with doxycycline (0.5 μg/ml) for 7 days and stained with crystal violet. **C,** Overexpression of Pdcd4(1-156) reduces cell proliferation. H1299 cells expressing Dox-inducible Pdcd4(1-156) or Pdcd4(1-156)(3A) were seeded onto a 96-well plate and treated with doxycycline (0.5 μg/ml) for 2- or 4-day, and then analyzed using XTT assay. The absorbance of control cells on day 0 was set to 1. Data was analyzed using a two-sample *t*-test (n=4; mean±s.d.; *P < 0.05; ***P < 0.001). **D,** PFKFB3 reverses the proliferation inhibition by Pdcd4(1-156). H1299 cells harboring Pdcd4(1-156) co-expression with or without PFKFB3 were seeded onto a 96-well plate, and analyzed using XTT assay. Left panel, expression levels of Pdcd4(1-156) and PFKFB3; Right panel, relative cell growth of control, Pdcd4(1-156), and Pdcd4(1-156) plus PFKFB3 expressing cells. The absorbance of control cells on day 0 was set to 1. Data were analyzed by a two-sample *t*-test (n=4; mean±s.d.; ***P < 0.001). **E, F, and G,** Pdcd4(1-156) attenuates tumor growth *in vivo*. H1299 cells expressing Dox-inducible empty vector (control), Pdcd4(1-156), and Pdcd4(1-156)(3A) were subcutaneously injected into nude mice. One day after injection, the mice were orally administered doxycycline (5 mg/kg) daily for 36 days. E, Tumor growth curves. Tumor volumes were measured using a caliper, and the volumes at day 37 were analyzed by a two-sample *t*-test (n=6; mean±s.d.; ***P < 0.001). F, Image of tumors after administration of doxycycline for 36 days. G, Tumor weights on day 37 were analyzed by a two-sample *t*-test (n=6; mean±s.d.; ***P < 0.001). **H,** Pdcd4(1-156) decreased proliferation in tumors. The expressions of Ki-67 (proliferation), cleaved caspase 3 (apoptosis), PFKFB3, and Pdcd4(1-156) in tumors were examined by IHC staining with antibody against Ki-67, cleaved caspase3, PFKFB3, and HA, respectively. The representative images are shown. Bar: 110 μm.

To examine the impact of Pdcd4-Rictor interaction on the growth of NSCLC cells, we generated the Dox-inducible HA-tagged Pdcd4(1-156) and Pdcd4(1-156)(3A) H1299 cell lines using a lentivirus-mediated expression system. Treatment with 0.5 μg/ml doxycycline robustly induced the expression of both Pdcd4(1-156) and Pdcd4(1-156)(3A), as confirmed by immunoblotting (Fig. 5A). Cells expressing the control vector, Pdcd4(1-156), or Pdcd4(1-156)(3A) were treated with doxycycline (0.5 μg/ml) and simultaneously subjected to a colony formation assay for 7 days. The results revealed that H1299 cells expressing Pdcd4(1-156) had a significant decrease in colony formation compared to the cells expressing empty vector (control) and Pdcd4(1-156)(3A) (Fig. 5B). In agreement with these findings, Dox-induced expression of Pdcd4(1-156) significantly reduced cell proliferation compared to Pdcd4(1-156)(3A) or empty vector controls (Fig. 5C). To define the role of PFKFB3 in the proliferation suppression mediated by Pdcd4(1-156), H1299 cells were transiently transfected with Pdcd4(1-156) and PFKFB3 expression plasmids. As shown in Fig. 5D, Pdcd4(1-156) expression slowed cells proliferation relative to the empty vector controls, mirroring the effect observed in Dox-inducible cells in Fig. 5B. This inhibitory effect on proliferation was reversed by co-expression of PFKFB3, indicating that Pdcd4(1-156)-mediated downregulation of PFKFB3 contributes to the proliferation suppression (Fig. 5D).

To validate the *in vitro* findings, a tumor xenograft model derived from H1299 cells was used to assess the effect of Pdcd4-Rictor interaction on tumor growth *in vivo*. Dox-inducible cells were subcutaneously injected into nude mice, with doxycycline (5 mg/kg) administration beginning one day post-injection. Tumors derived from Pdcd4(1-156) expressing cells grew significantly slower than those derived from Pdcd4(1-156)(3A)-expressing or control vector cells (Fig. 5E). At day 37, the average tumor volume of Pdcd4(1-156)-expressing tumors was reduced to approximately 20-25% of that observed in Pdcd4(1-156)(3A)-derived or control tumors (P = 1.8 X 10^−5^ and P = 2.5 X 10^−5^, respectively). Similarly, the average tumor weight of Pdcd4(1-156)-expressing tumors decreased to about 25-30% of that of Pdcd4(1-156)(3A)-derived tumors or control tumors (P = 1.1 X 10^−4^ and P = 6.8 X 10^−5^, respectively) (Figs. 5F and G). To further confirm that expression of Pdcd4(1-156) reduced proliferation *in vivo*, tumor sections were subjected to immunohistochemical staining with an antibody against the proliferation maker Ki-67. Tumors derived from Pdcd4(1-156)-expressing cells exhibited the lowest Ki-67-positive staining compared to tumors derived from Pdcd4(1-156)(3A)-expressing cells or empty-vector controls (Fig. 5H). Conversely, staining of cleaved caspase-3, a marker for apoptosis, was most prominent in tumors with Pdcd4(1–156) expression. These findings suggest that Pdcd4-Rictor interaction leads to decreased tumor growth. Furthermore, supporting our *in vitro* data that Pdcd4(1-156) facilitates PFKFB3 degradation, PFKFB3 staining was markedly reduced in tumors expressing Pdcd4(1-156) compared to those with Pdcd4(1-156)(3A) or empty vector (Fig. 5H).

### Protein levels of Rictor and PFKFB3 are positively correlated in NSCLC patients

**Fig. 6:**
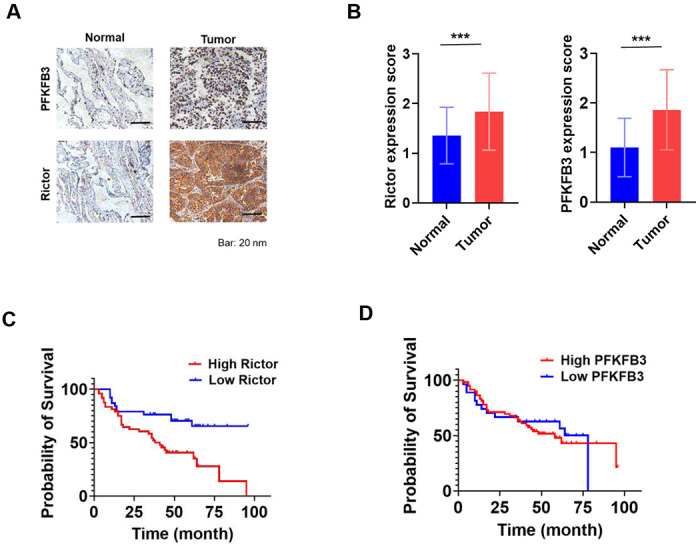
Rictor and PFKFB3 levels are increased in NSCLC patients. **A,** Representative IHC images of Rictor and PFKFB3 in normal and tumor lung tissues. **B,** Rictor and PFKFB3 protein levels are elevated in cancerous tissues. Data were analyzed by the Wilcoxon matched-pairs signed rank test (n=87, mean±s.d.; ***P < 0.001). **C,** Patients with high Rictor protein levels show lower survival rates. The Kaplan-Meier curves and log-rank test were used to compare the overall survival between patients with high and low Rictor protein expression (*P* = 0.0019). **D,** No association between overall survival and PFKFB3 protein expression. Data was analyzed using Kaplan-Meier curve and log-rank test (*P* = 0.8129).

Knockdown of Rictor decreased PFKFB3 protein abundance (Fig. 4H), suggesting that Rictor plays a role in regulating PFKFB3 expression. To explore the clinical significance of Rictor and PFKFB3 expression, we analyzed the lung adenocarcinoma datasets from The Cancer Genome Atlas PanCancer study and OncoSG study. Our analysis revealed no correlation between *rictor* mRNA and *pfkfb3* mRNA levels in both datasets (Supplementary Fig. 3). However, protein expression data of PFKFB3 were not available in these datasets. To investigate the relationship between Rictor and PFKFB3 protein levels in NSCLC, we performed immunohistochemical analysis using the NSCLC TMA. TMA slides containing 87 NSCLC samples and their matched adjacent normal tissues were stained with antibodies against Rictor or PFKFB3, showing positive protein expression as brown staining and nuclei as blue with hematoxylin counterstain (Fig. 6A).

The expression levels of Rictor and PFKFB3 in tumor and normal lung tissues were blindly evaluated by a pathologist using a standardized scoring system taking into account both percentage of cells staining and staining intensity. Scores ranged from 0 to 3: 0 indicates no staining; 1+ denotes dim staining in any tumor cells, or moderate intensity staining in less than 1/3 cells of interest; 2+ corresponds to moderate intensity staining in greater than 1/3 cells of interest; 3+ signifies strong staining in greater than 2/3 cells of interest. Regarding Rictor staining, 60 cases (69%) of normal tissues displayed weak expression, 23 cases (26%) showed moderate expression, and only 4 cases (5%) had strong expression (Table I). In comparison, among tumor tissues, 55 cases (63%) demonstrated moderate to strong Rictor staining, while the remaining 32 cases (37%) exhibited weak or no staining. A significant increase in Rictor expression was observed in tumor samples compared with adjacent normal tissues, as determined by the Wilcoxon matched-pairs signed rank test (*P* < 0.0001) (Fig. 6B). Regarding PFKFB3 expression in normal tissues, 69 cases (79%) exhibited none to weak staining, while moderate to strong staining was detected in 18 cases (21%) (Table I). In contrast, analysis of tumor tissues revealed that 27 cases (31%) had none or weak PFKFB3 staining, whereas 50 cases (57%) displayed moderate to strong staining. PFKFB3 expression was significantly higher in tumors compared with adjacent normal tissues (*P* < 0.0001) (Fig. 6B). In tumor samples, Rictor expression was found to be weakly but significantly correlated with PFKFB3 expression (*r* = 0.2135; *P* = 0.047). However, when comparing cases with high (strong and moderate) versus low (weak and none) expression of Rictor or PFKFB3, there were no significant differences in clinical or pathological characteristics among the patient groups.

Notably, NSCLC patients with elevated Rictor protein levels had lower overall survival compared to patients with lower levels of Rictor protein, as assessed by Kaplan-Meier analysis and log-rank test (*P* = 0.0019) (Fig. 6C). However, there was no statistically significant difference in overall survival between patients with high and low PFKFB3 expression (*P* = 0.8129) (Fig. 6D). These clinical data reinforce the results observed in cell culture and mouse models (Fig. 4), demonstrating that Pdcd4/mTORC2 axis reduces PFKFB3 protein abundance and low Rictor expression is associated with improved survival rate.

## DISCUSSION

Our previous work established that Pdcd4 suppresses mTORC2 activity by inhibiting the translation of Sin1 [21]. Pdcd4 functions as a translational inhibitor by binding to eIF4A and blocking its helicase activity, thereby reducing *sin1* mRNA translation and ultimately impeding invasion. In addition to translation-dependent tumorigenesis suppression, here, we demonstrate that Pdcd4 can also suppress tumorigenesis through a translation-independent mechanism. Particularly, we show that Pdcd4 directly binds with Rictor, preventing its association with mTOR and thereby disrupting the formation of the mTORC2 complex. Consequently, mTORC2 kinase activity is impaired (Fig. 3), resulting in reduced tumor cell growth both *in vitro* and *in vivo* (Fig. 5). Our mapping studies identified amino acid residues R105, K108, and R110 are required for Pdcd4 binding to Rictor (Fig. 2F). Notably, R110 was reported to undergo methylation by PRMT5, which is linked to enhanced cell viability under nutrient deprivation [38, 41]. Our results showed that methylated Pdcd4(1-156) and Me-RBD peptide abolished the Rictor binding ability (Fig. 2I and Supplementary Fig. 1), suggesting that PRMT5-mediated methylation of R110 weakens the Pdcd4-Rictor interaction and thereby enhances mTORC2 activity. Since mTORC2 phosphorylates its downstream targets, such as Akt, PKCα, and SGK1, it drives the expression and activation of pro-survival genes and proteins, as well as metabolic reprogramming to support cell proliferation and survival [42]. Thus, reduced Pdcd4-Rictor interaction due to methylation can facilitate tumor cell growth and survival. This is further supported by our current findings: (i) the growth rate of Pdcd4(1–156)-expressing cells was significantly slower than those expressing the Rictor-binding-deficient mutant Pdcd4(1–156)(3A) in both cultured cells and mice (Figs. 5E–G), and (ii) immunohistochemical analysis revealed lower Ki-67 and higher cleaved caspase-3 staining in Pdcd4(1–156)-derived tumors compared to Pdcd4(1–156)(3A)-derived tumors (Fig. 5H).

Our data indicate that Pdcd4, Pdcd4(1-156), and the RBD peptide specifically disrupt mTORC2 complex formation without impacting mTORC1 assembly (Figs. 3A–C). In addition, overexpression of Pdcd4(1-156) significantly inhibited Akt phosphorylation at Ser473, a marker of mTORC2 activity, while leaving S6K phosphorylation at Thr389, a target of mTORC1, unchanged (Figs. 3D and E). These findings demonstrate that Pdcd4(1-156) selectively inhibits mTORC2 while sparing mTORC1 function. Rictor amplification has been recognized as a driver of genetic alternation in about 8-13% of NSCLC [43, 44]. Clinical evidence suggests that a lung cancer patient with Rictor-amplification responds more favorably to dual mTORC1/mTORC2 inhibitors compared to other chemotherapy drugs [44]. However, suppression of mTORC1 can trigger feedback activations of cell survival and metastasis pathways [45], potentially responsible for tumor relapses following dual mTORC1/C2 inhibitor treatment in the index lung cancer patient [44]. Indeed, treating cells with the mTORC1/C2 inhibitor, ADZ2014, has been shown to activate the ERK survival pathway [46]. Taken together, these findings highlight that selective inhibition of mTORC2, while preserving mTORC1 activity, could offer a more effective therapeutic strategy for patients with Rictor-elevated lung cancer. Our results showing that Pdcd4(1-156) and RBD peptide specifically inhibit mTORC2, but not mTORC1, point to a potential new approach for the development of mTORC2 specific inhibitors for NSCLC with Rictor amplification.

An important aspect of our findings is that the Pdcd4-Rictor interaction also regulated glycolysis through modulation of PFKFB3 protein abundance (Fig. 4). The mTORC2/Akt signaling axis is well-established in controlling glycolytic flux by regulating the activation and expression of several proteins involved in glycolysis [11]. Our data demonstrated that either overexpression of Pdcd4(1-156) or knockdown of Rictor led to decreased PFKFB3 protein levels (Figs. 4B and C), which indicates mTORC2 regulating PFKFB3 expression. PFKFB3 is a key glycolytic regulator, controlling the activation of the rate-limiting enzyme, PFK-1 [14]. Notably, PFKFB3 is frequently up-regulated in lung cancer [15]. Increased levels of PFKFB3 accelerate glycolysis, which not only enhances ATP production but also generates metabolic intermediates required for nucleotides, lipids, and amino acid biosynthesis to support rapidly proliferating tumor cells [47]. Inhibition or down-regulation of PFKFB3 has been shown to reduce cell proliferation and induce apoptosis in lung cancer cells and suppresses tumor growth in nude mice [16]. Therefore, our observation that Pdcd4-mediated reduction of PFKFB3 protein abundance is likely to contribute to the tumor-suppressive effects in both *in vitro* and *in vivo* models. Mechanistically, the selective attenuation of PFKFB3 protein abundance by Pdcd4(1-156) is reversed upon treatment with the proteasome inhibitor MG132 (Fig. 4G), suggesting that Pdcd4(1-156) promotes proteasome-mediated degradation of PFKFB3. This is further supported by the increased ubiquitination of PFKFB3 observed in Pdcd4(1-156)-expressing cells, but not in those expressing Pdcd4(1-156)(3A) or empty vector controls (Fig. 4I). In agreement with these findings, our analysis of NSCLC datasets revealed no correlation between *rictor* and *pfkfb3* mRNA levels (Supplementary Fig. 3), while immunohistochemical analysis of NSCLC TMA showed a positive correlation between their protein levels (Table I). Previous studies have reported that PFKFB3 can be ubiquitinated by the E3 ligase APC/C-Cdh1 at KEN box and SCFβ-TrCP at DSG box [40, 48]. The protein level of PFKFB3 is tightly regulated during cell cycle progression, with APC/C-Cdh1 activity predominating in mid-to-late G1 phase and SCF-β-TrCP becoming active during S phase. However, it is unclear whether the APC/C-Cdh1, SCF-β-TrCP, or an unidentified E3 ligase is responsible for PFKFB3 ubiquitination in the context of Pdcd4/Rictor-mediated regulation, warranting further investigation. Additionally, the precise molecular mechanism by which Pdcd4/Rictor promotes PFKFB3 ubiquitination and degradation is not yet defined and requires further study.

In summary, our study uncovers a novel mechanism by which Pdcd4 suppresses tumorigenesis in NSCLC through direct binding with Rictor, resulting in selective inhibition of mTORC2 activity and subsequent degradation of PFKFB3 via the ubiquitin-proteasome pathway. Importantly, Pdcd4(1-156) and RBD peptide specifically inhibit the activity of mTORC2 without affecting mTORC1, providing a new potential direction for designing mTORC2-specific inhibitor for treatment of Rictor-amplified NSCLC.

## Supplementary Material

This is a list of supplementary files associated with this preprint. Click to download.

- Table1.docx
- Supplementarymaterials.docx

## Figures and Tables

**Fig. 7:**
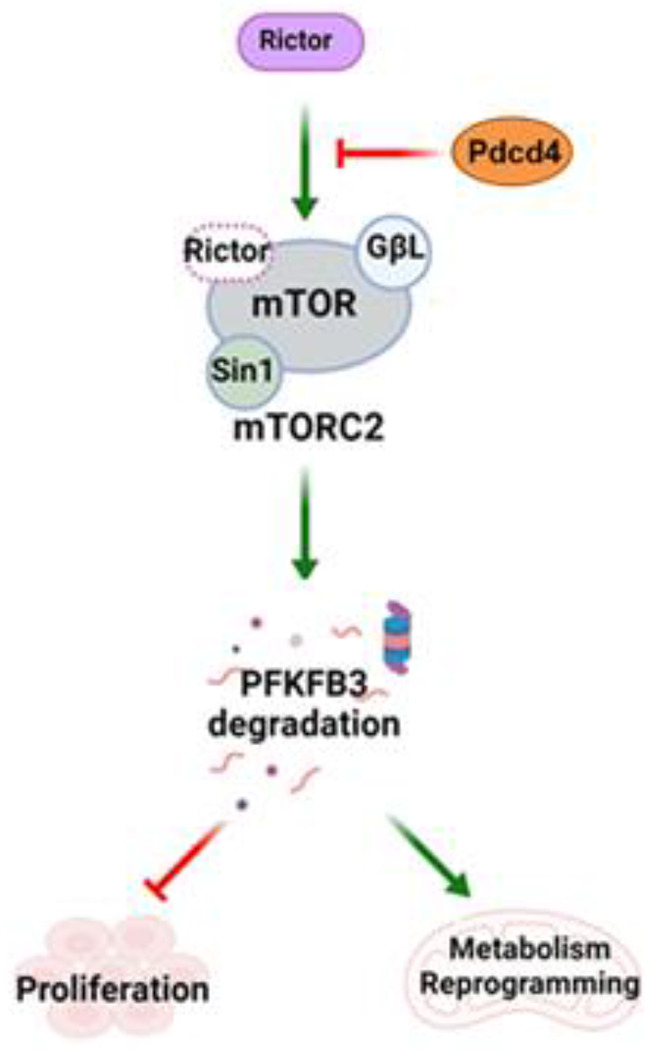
The schematic diagram illustrates the mechanism by which Pdcd4 suppresses tumorigenesis via its interaction with Rictor. When Pdcd4 binds to Rictor, it prevents the subsequent association of Rictor with mTOR, resulting in diminished mTORC2 activity. Consequently, this process facilitates the degradation of the key glycolytic enzyme PFKFB3, leading to metabolic reprogramming and inhibition of cell proliferation and tumor growth.
